# Activity-dependent conformational transitions of the insulin receptor–related receptor

**DOI:** 10.1016/j.jbc.2021.100534

**Published:** 2021-03-11

**Authors:** Oleg V. Batishchev, Natalia V. Kuzmina, Andrey A. Mozhaev, Alexander S. Goryashchenko, Ekaterina D. Mileshina, Alexander N. Orsa, Eduard V. Bocharov, Igor E. Deyev, Alexander G. Petrenko

**Affiliations:** 1 A.N. Frumkin Institute of Physical Chemistry and Electrochemistry, Russian Academy of Sciences, Moscow, Russia; 2 Shemyakin–Ovchinnikov Institute of Bioorganic Chemistry, Russian Academy of Sciences, Moscow, Russia; 3 Shubnikov Institute of Crystallography of Federal Scientific Research Centre “Crystallography and Photonics”, Russian Academy of Sciences, Moscow, Russia; 4 Moscow Institute of Physics and Technology, Dolgoprudniy, Moscow Region, Russia

**Keywords:** insulin receptor, protein tyrosine-kinase (tyrosine kinase), atomic force microscopy, protein structure, conformational transitions, insulin receptor–related receptor, receptor structure function, autophosphorylation, insulin, AM, atomic force microscopy, cryo-EM, cryo-electron microscopy, DDM, n-Dodecyl β-D-maltoside, IGF-1R, insulin-like growth factor 1 receptor, IR, insulin receptor, IRR, insulin receptor-related receptor, RTK, receptor tyrosine kinase

## Abstract

The insulin receptor (IR), insulin-like growth factor 1 receptor (IGF-1R), and insulin receptor-related receptor (IRR) form a mini family of predimerized receptor-like tyrosine kinases. IR and IGF-1R bind to their peptide agonists triggering metabolic and cell growth responses. In contrast, IRR, despite sharing with them a strong sequence homology, has no peptide-like agonist but can be activated by mildly alkaline media. The spatial structure and activation mechanisms of IRR have not been established yet. The present work represents the first account of a structural analysis of a predimerized receptor-like tyrosine kinase by high-resolution atomic force microscopy in their basal and activated forms. Our data suggest that in neutral media, inactive IRR has two conformations, where one is symmetrical and highly similar to the inactive Λ/U-shape of IR and IGF-1R ectodomains, whereas the second is drop-like and asymmetrical resembling the IRR ectodomain in solution. We did not observe complexes of IRR intracellular catalytic domains of the inactive receptor forms. At pH 9.0, we detected two presumably active IRR conformations, Γ-shaped and T-shaped. Both of conformations demonstrated formation of the complex of their intracellular catalytic domains responsible for autophosphorylation. The existence of two active IRR forms correlates well with the previously described positive cooperativity of the IRR activation. In conclusion, our data provide structural insights into the molecular mechanisms of alkali-induced IRR activation under mild native conditions that could be valuable for interpretation of results of IR and IGF-IR structural studies.

Receptor tyrosine kinases (RTKs) represent a vitally important superfamily of transmembrane cellular proteins functioning as receptors for a large variety of growth factors and hormones (1). RTKs constitute more than 60% of the protein tyrosine kinase family of the human genome (2), and their mutations, malfunctions, and aberrant activations lead to severe diseases such as cancer, diabetes, arteriosclerosis, etc. (3). Activation of RTKs often requires dimerization and oligomerization of the receptor upon ligand binding (1). However, a small set of RTKs, representing the most commonly expressed insulin receptor (IR) and accordingly named the insulin receptor family, preexists in the membrane as disulfide-linked dimers of two subunits, called αβ half-receptors and, thus, requires different mechanisms of activation and signal transduction across the plasma membrane (4). Along with IR, this mini family contains the highly homologous insulin-like growth factor 1 receptor (IGF-1R) and the insulin receptor–related receptor (IRR) (4). These proteins have a similar domain structure comprising N-terminal extracellular regions with two leucine-rich repeat domains, named L1 and L2, joined by the cysteine-rich C-domain and three C-terminal fibronectin type III repeats (FnIII-1, FnIII-2, and FnIII-3). The single-pass transmembrane hydrophobic segment of these proteins is followed by a flexible cytoplasmic juxtamembrane region connecting the C-terminal intracellular catalytic tyrosine kinase domain (5).

Intensive studies of IR and IGF-1R by structural methods such as X-ray crystallography, cryo-electron microscopy (cryo-EM), and small-angle X-ray scattering reveals details of ligand binding and subsequent protein remodeling (6, 7, 8, 9, 10, 11, 12, 13). Both receptors show inactive Λ-shape of the ectodomain region transforming into active predominantly T-like conformation for IR or Γ-shape of IGF-1R upon interaction with insulin or IGF-1, respectively. This activation requires significant structural rearrangements in both ectodomain and transmembrane parts of the proteins allowing the tyrosine kinase activation and subsequent phosphorylation of C-terminal tyrosines of both receptor subunits.

IRR, discovered in 1989, is an orphan receptor, which has no proteinaceous endogenous ligands (14). It is not as abundant as IR and is primarily expressed in cell subsets of kidneys, stomach, and pancreatic tissues (15). It has been recently shown that IRR alkali-induced activation is dose-dependent, quick, and reversible; thus, IRR can serve as a cellular regulator of acid-base equilibrium (16). This activation triggers typical for RTKs processes of autophosphorylation and subsequent phosphorylation of intracellular signaling proteins insulin receptor substrate 1 and AKT1 (Protein kinase B) along with actin cytoskeleton rearrangements (16, 17, 18).

Ability to react to an alkaline media is a feature unique to IRR as neither IR nor IGF-1R can be activated by either alkali or acid. Although IRR knockouts in mice are not as fatal as those of IR or IGF-1R, they lead to failures in renal function and behavioral abnormalities under experimental alkalosis (19, 20).

Recently, we have obtained the structure of the IRR ectodomain by means of small-angle X-ray scattering and atomic force microscopy (AFM) at both alkaline and neutral pH conditions (21). We demonstrated that IRR ectodomain, unlike Λ-shape of IR, adopts asymmetrical dimeric drop-like conformation with no detectable differences between pH 7.0 and pH 9.0. Similar drop-like conformation of the ectodomain was recently detected for the ligand-bound active form of IGF-1R (12). This conformation shows a small distance between the fibronectin domains of the two receptor subunits indicating that FnIII-2 and FnIII-3 domains are most critical for IRR activity. This observation is supported by mutant analyses of IRR (17). The drop-like shape of the IRR ectodomain is also supported by the mutagenesis data suggesting a key importance of five conserved amino acid residues (17) of the L1C domains for the IRR activity. In this model, they are located at the interface between the L1C and fibronectin domains within the IRR dimer that explains their role in the IRR function and implies that L1C domains are components of the pH sensing mechanism of the IRR (21).

Similar shapes of the IRR ectodomain at both pH 7.0 and pH 9.0 point to a possible role of juxtamembrane receptor regions in its pH sensing and large-scale protein rearrangements upon activation. Clarification of this hypothesis requires obtaining structural data on the full-length protein containing transmembrane and tyrosine kinase domains. Here, we utilized AFM to elucidate structural peculiarities of the full-length IRR in detergent micelles. AFM is widely used to get information on ligand–receptor interactions (22), protein-membrane interactions (23, 24), and a shape of membrane proteins (22). Unlike cryo-EM or X-ray crystallography, AFM allows studying proteins in their native environment in solution, preserving their fully hydrated and movable state, as well as in lipid environment in nanodiscs (23, 24, 25, 26).

Proper hydration is extremely valuable for investigation of glycosylated proteins, such as IRR. Usage of lipid nanodiscs or detergent micelles allows imaging of transmembrane proteins, providing necessary environment for hydrophobic membrane parts of these proteins (26). Our data demonstrate how AFM can be adopted for in-depth structural studies of heterogeneous transmembrane receptors of type I such as RTKs and reveal a set of possible conformations of IRR.

## Results

### IRR expression, purification, and autophosphorylation *in vitro*

**Figure 1:**
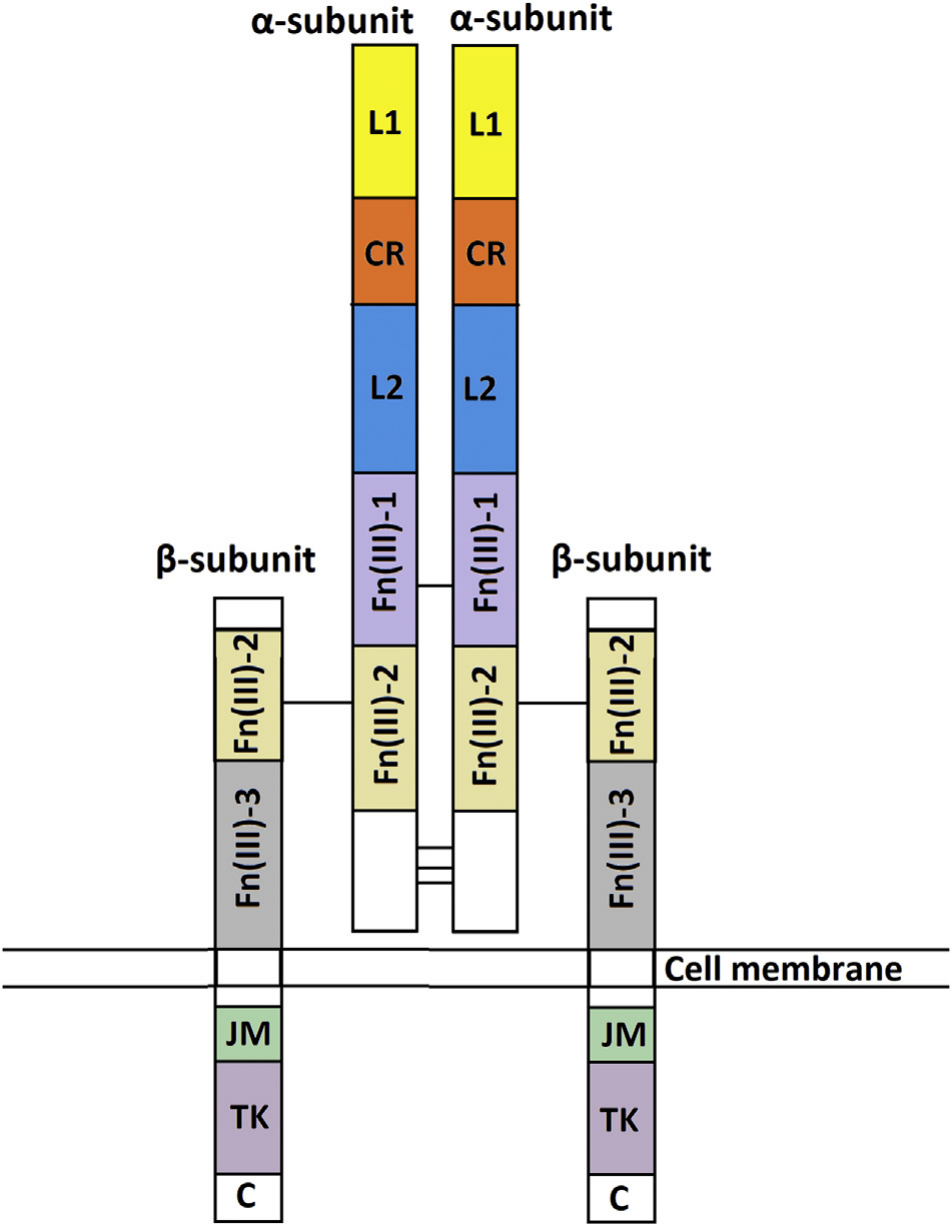
**Schematic representation of the domain organization of the IRR.** L1, the first leucine rich domain; CR, the cysteine rich domain; L2, the second leucine rich domain; Fn(III)-1, the first fibronectin type III domain; Fn(III)-2, the second fibronectin type III domain; Fn(III)-3, the third fibronectin type III domain; C – C-terminus, disulfide bonds are shown as *black lines*. IRR, insulin receptor-related receptor; JM, junctions membrane domain; TK, the tyrosine kinase catalytic domain.

The best way to express eukaryotic glycoproteins that have a complex structure was to use mammalian cells that made it possible to retain the native tertiary structure and posttranslational modifications, such as glycosylation. Plasmid vector pcDNA-IRR-HIS containing all necessary elements for the successful expression of the target membrane protein of IRR was obtained. The full-length IRR (Fig. 1) was produced by human embryonic kidney cell-derived cells.

Next, transient transfection of HEK293 cells with the pcDNA-IRR-HIS vector was performed. Cells were grown on Dulbecco's modified Eagle's medium culture medium; for large-scale production, Eppendorf cell culture flasks T175 were used. The full-length IRR was produced as a single-chain precursor (about 170 kDa) that undergoes proteolytic processing to generate the mature disulfide linked heterotetrameric receptor of two α (100 kDa each) and two β (70 kDa each) subunits. Confirmation of the enzymatic activity of the receptor was carried out in cells before further extraction and purification. To extract IRR, the cells were solubilized in n-Dodecyl β-D-maltoside (DDM) at a final concentration of 1% (w/v). For this detergent, the possibility of maintaining a high level of kinase activity for similar receptor tyrosine kinases has been shown (27, 28, 29).

After solubilization, IRR was purified with a combination of Ni Sepharose 6 Fast Flow and Cyanogen bromide-activated-Sepharose 4B with linked monoclonal antibodies 4D5. These antibodies were described earlier (30, 31).

**Figure 2:**
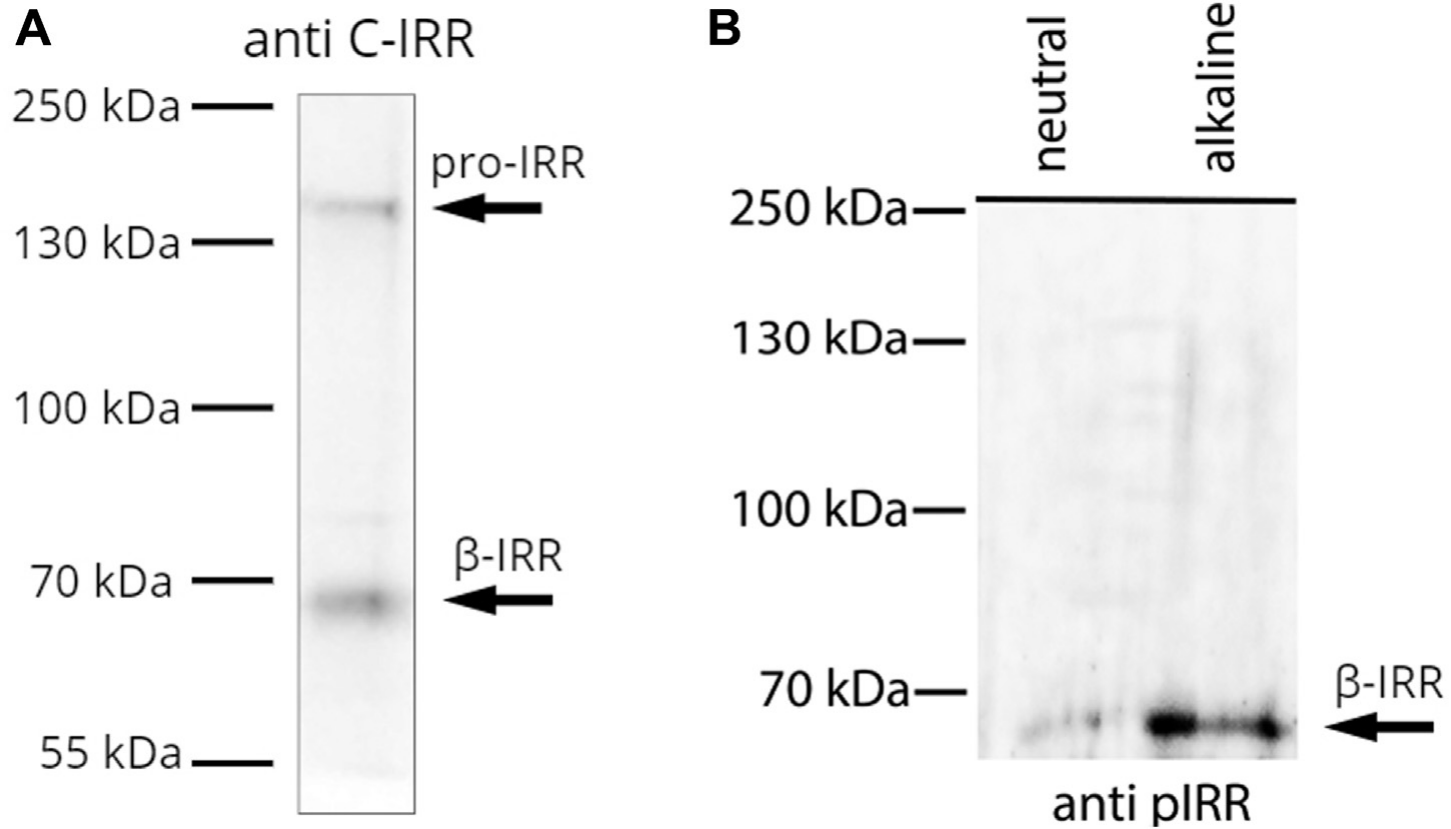
**Western blot analysis of IRR.***A,* Western blot detection of IRR after purification using the β-subunit of IRR antibodies (anti C-IRR), with addition of β-mercaptoethanol. pro-IRR is an uncleaved IRR proreceptor. To the left, the weights of protein markers (in kDa) are given. *B*, *In vitro* phosphorylation of IRR. Activation of IRR by alkali in the autophosphorylation assay. Transiently expressed IRR was purified and activated by Tris-HCl buffer, pH 7.4 or 9.0. Samples were blotted with antibodies against phosphorylated form of IRR (anti pIRR). To the left, the weights of protein markers (in kDa) are given. IRR, insulin receptor-related receptor.

The expression level and the purification steps were monitored using the Western blot analysis. To confirm IRR expression, the cell lysates were stained with rabbit anti-C-IRR antibodies against the β-subunit of the receptor (16) (Fig. 2*A*). The blot showed two bands: the β-subunit itself (70 kDa) and uncleaved IRR precursor (170 kDa). IRR was further tested by the *in vitro* autophosphorylation assay. The samples were incubated with Tris-HCl buffer of a neutral or alkaline pH and further analyzed by blotting with antibodies against phosphorylated forms of IRR (anti-pIRR) (Fig. 2*B*).

### Atomic force microscopy of IRR

**Figure 3:**
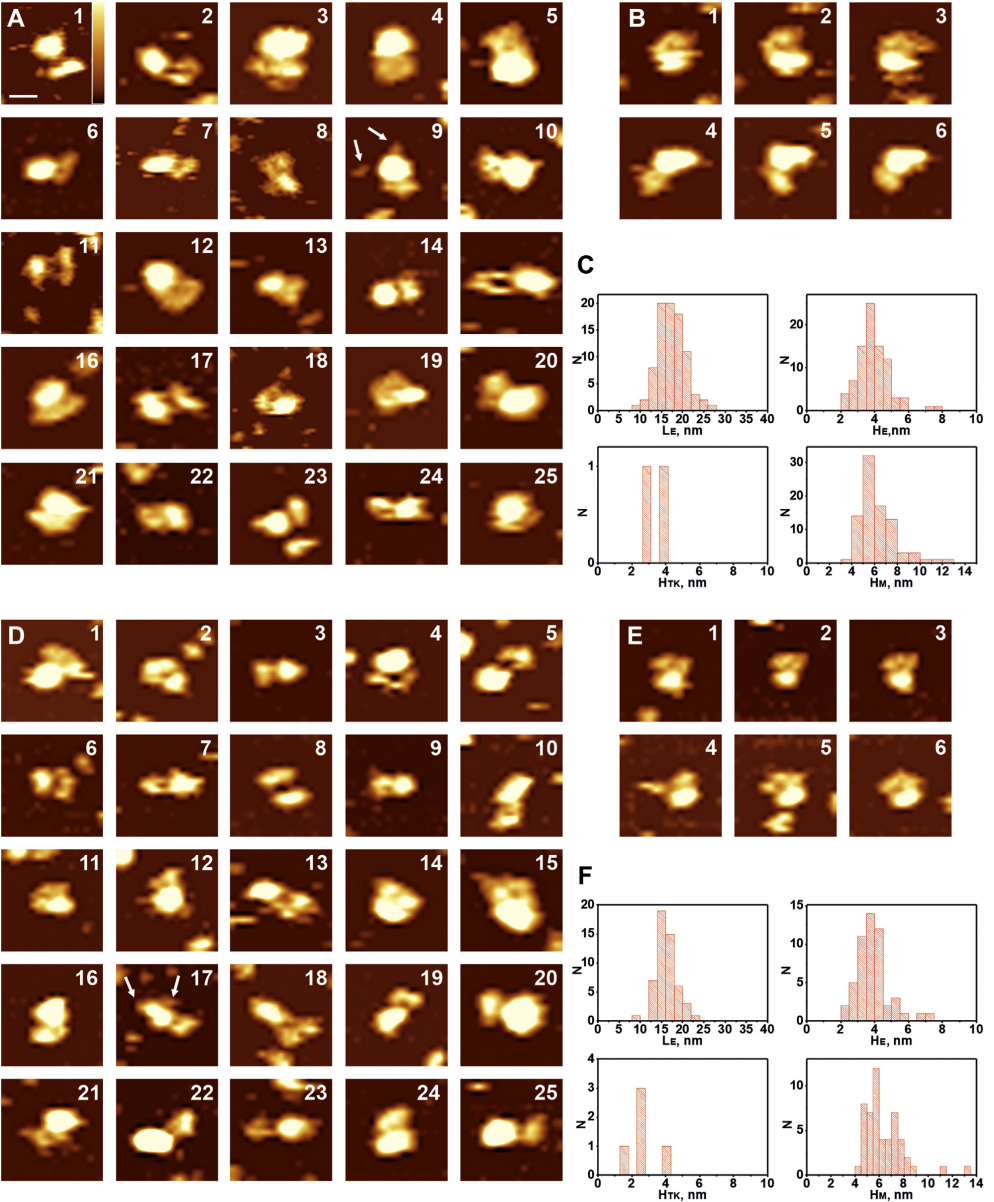
**AFM images of the full-length IRR in micelles at pH 7.0 (*upper part*, *A–C*) and pH 9.0 (*lower part*, *D–F*) in working buffer solution as described in Experimental procedures section with the U-shape of the ectodomain**. *White arrows* point to the structure of tyrosine kinase domains (in A9, D12, D17). *A* and *D*, sets of images for the U-shape of the IRR. *B* and *E*, sequential scans (1–3 and 4–6) of the same full-length IRR molecule in micelle. *C* and *F*, histograms representing distributions of lengths of the ectodomain (L_E_), heights of the ectodomain (H_E_), heights of the tyrosine kinase domain (H_TK_), and heights of micelles with the transmembrane part of the IRR (H_M_). Scale bar is 20 nm, Z range (color-coded) is 4 nm (shown in image A1). AFM, atomic force microscopy; IRR, insulin receptor-related receptor.

**Figure 4:**
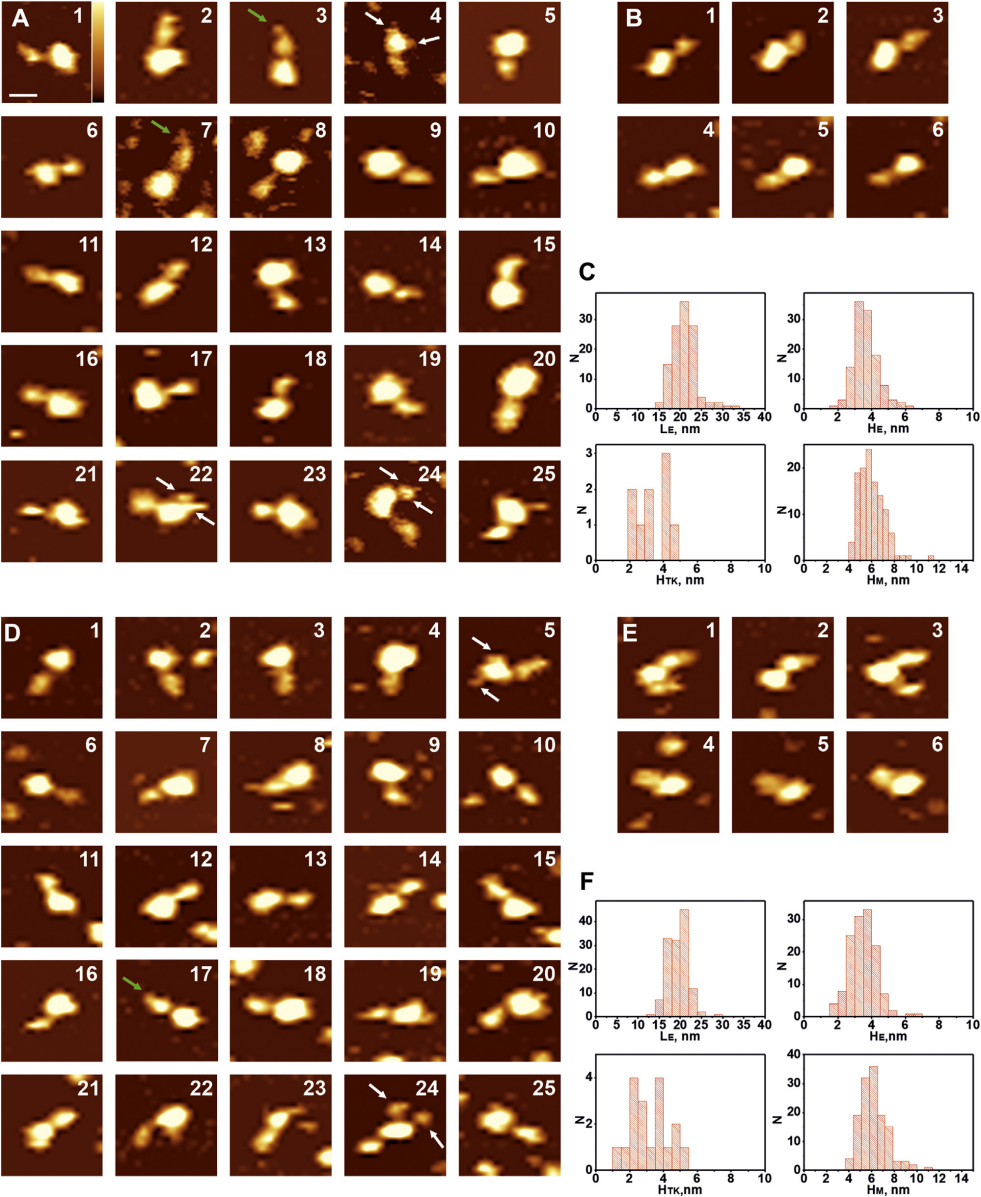
**AFM images of the full-length IRR in micelles at pH 7.0 (*upper part*, *A–C*) and pH 9.0 (*lower part*, *D–F*) in the working buffer solution, as described in Experimental procedures section, with the drop-like shape of the ectodomain**. *White arrows* point to the structure of tyrosine kinase domains (in A4, A22, A24, D5, D24), *green arrows* indicate L1-CR region in extended conformations (in A3, A7, D17). *A* and *D*, sets of images for the drop-like shape of the IRR. *B* and *E*, sequential scans (1–3 and 4–6) of the same full-length IRR molecule in micelle. *C* and *F*, histograms representing distributions of lengths of the ectodomain (L_E_), heights of the ectodomain (H_E_), heights of the tyrosine kinase domain (H_TK_), and heights of micelles with the transmembrane part of the IRR (H_M_). Scale bar is 20 nm, Z range (color-coded) is 4 nm (shown in image A1). AFM, atomic force microscopy; IRR, insulin receptor-related receptor.

**Figure 5:**
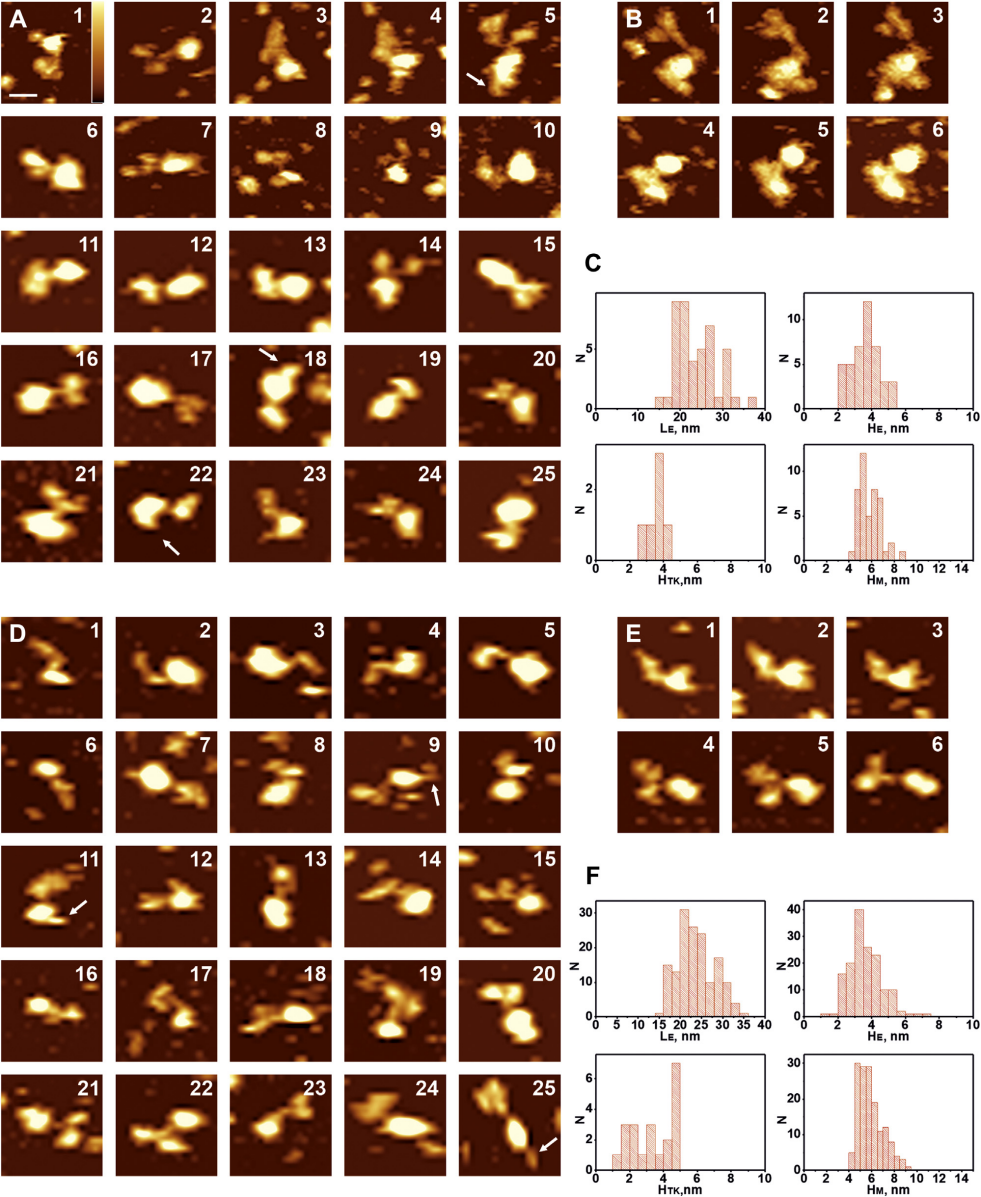
**AFM images of the full-length IRR in micelles at pH 7.0 (upper part, A–C) and pH 9.0 (lower part, D–F) in working buffer solution, as described in Experimental procedures, with the T- and Γ-shapes of the ectodomain**. *White arrows* point to the structure of tyrosine kinase domains (in A5, A18, A22, D9, D11, D25). *A* and *D*, set of images for the T- and Γ-shapes of the IRR. *B* and *E*, two sequential scans (1–3 and 4–6) of the same full-length IRR molecule in micelle. *C* and *F*, histograms representing distributions of lengths of the ectodomain (L_E_), heights of the ectodomain (H_E_), heights of the tyrosine kinase domain (H_TK_), and heights of micelles with the transmembrane part of the IRR (H_M_). Scale bar is 20 nm, Z range (color-coded) is 4 nm (shown in image A1). AFM, atomic force microscopy; IRR, insulin receptor-related receptor.

We performed an AFM study, in which IRR was incorporated into DDM micelles and then deposited on the mica surface, to which it bound via electrostatic and van der Waals interactions. Scanning was performed in the working buffer solution of 150 mM NaCl, 20 mM sodium phosphate, and 5% glycerol (pH 7.0 or 9.0) in tapping mode using ultrasharp cantilevers with the tip radius of about 2 nm to get a resolution close to that of cryo-EM technique. In contrast to imaging of transmembrane proteins embedded in supported lipid bilayer, scanning of protein micelles gives us information about structures of both extracellular and tyrosine kinase parts of the molecule. Obtained images are heterogeneous, representing a lot of protein conformations, aggregates, as well as empty micelles (Fig. S1). Among them, micelles are the most uniform and detectable. Based on this observation, we postulated the following criteria to select the proper IRR molecules in DDM micelles: (i) height of the single IRR-containing micelle should not be less than 4.5 nm (32) that corresponds to the size of two DDM molecules and the transmembrane part of the IRR between them and (ii) upon sequential scanning, protein–micelle complexes should remain connected. The latter condition allows excluding moving particles from the images, which are not part of IRR molecules. Such particles shift away from the protein molecules by lateral forces of tapping mode and thermal drift. Thus, we performed more than 100 scans repeating each at least three times, with 5 min intervals between starts of each sequential scan. On average, we observed 100 to 200 micelles and protein–micelle complexes in each scan; thus, more than 10,000 particles were analyzed. Among these micelles and protein–micelle complexes, we detected 76% of empty micelles at pH 7.0 and 73% at pH 9.0 (Fig. S1). Focusing on single IRR-containing micelles, which satisfy the first criterion, we chose structures, which reflect possible IRR conformations. It should be noted that with the first criterion, we used height but not lateral dimensions of a micelle because the latter depends on a size of a cantilever tip and is always greater than the real size of the particle. Using the second criterion, we selected only those protein–micelle complexes, which have not lost connection between the parts upon sequential scanning (Figure 3, Figure 4, Figure 5, *B*, E). Finally, we obtained the typical conformations of the full-length IRR. These conformations represent all possible rotations of the protein–micelle complex in both lateral plane and around the big axis of the IRR ectodomain. For that reason, relative positions of the ectodomain and tyrosine kinase domains are not fixed with the latter located at different places around the surface projection of the micelle as well as above/below it. The same is observed for cryo-EM of the IR into lipid nanodiscs (8). The only restriction is that IRR ectodomains interact with the mica surface, which is the most widely used atomically flat support for AFM imaging. Mica possesses a negative charge in water solutions. It means that IRR cannot orient its negatively charged amino acid residues toward mica. Therefore, the number of the receptor’s possible orientations on the mica surface decreases. Additionally, positively charged residues can electrostatically bind to the surface, thus, preventing further rotations of IRR.

For the whole set of images obtained at pH 7.0, we observed U-shaped conformations of the IRR ectodomain with the distance between subunits of the ectodomain ranging from approximately 7 nm to 15 nm (Fig. 3, *A* and *D*). This was similar to inactive U-shape of the IGF-1R, which has the distance between subunits of 7 nm (11) or Λ-shaped conformation of the IR where such distance is about 12 nm (8). Tyrosine kinase domains in these conformations were bound to a micelle becoming mostly unresolved from it. This led to widening of the peak for the height distribution of the micelles that made it reach bigger values (Fig. 3, *C* and *F*). Length of the ectodomain in such conformation was (17 ± 3) nm, height—4 ± 1 nm (Fig. 3*C*). The same conformation was also observed at pH 9.0 with length of the ectodomain being 16 ± 2 nm and its height being 4 ± 1 nm (Fig. 3*F*). Figure 3, *B* and *E* represent examples of sequential scans of the same particle at pH 7.0 and 9.0, respectively.

It is noteworthy that both movements of the IRR domains and increase in lateral sizes due to AFM tip geometry influenced lateral sizes of the molecules. Thus, we did not provide width of the ectodomain focusing only on its length and height as the most reliable parameters.

The drop-like conformation of the IRR ectodomain above the micelle was the most abundant at both pH 7.0 and pH 9.0. (Fig. 4). This conformation was the only form of the soluble ectodomain which we observed earlier (21). At рН 7.0, length of the ectodomain in such conformation was 20 ± 3 nm, whereas height was 3.5 ± 0.5 nm (Fig. 4*C*), and the same values were yielded at рН 9.0 (Fig. 4*C*). These sizes correspond to those detected for soluble ectodomains (21). A flexible loop between L2 and CR domains allowed movement of the L1-CR region and formation of asymmetrically extended conformations (Fig. 4, images A3, A7, D17). However, such movement was rarely detected in pH 9.0. Tyrosine kinase domains were present at different mutual positions, and they moved at repeated scans representing structures from two distinct domains (Fig. 4, images A4, D5, D24) with separation distance between them being up to 20 nm from one part close to the micelle (Fig. 4, image A22). Their position on the top or below the micelle led to an increase in the micelle’s height detected by AFM because of the size of the tyrosine kinase domain (Fig. 4, *C* and *F*). This results in an appearance of a “tail” of about 2 to 4 nm on the right side of the peak at the height distribution histogram (Fig. 4, *C* and *F*) that exactly matches the size of tyrosine kinase domains (H_TK_). We did not observe any dependence of the domain dynamics on the direction of the tip movements. Thus, these movements are not likely to result from interactions with the tip.

In contrast to the individual soluble IRR ectodomain which only has a drop-like shape regardless of the pH of a medium for the full-length IRR in micelles, we detected other conformations of the receptor with structures resembling the active states of both IR and IGF-1R. Namely, we found asymmetrical and symmetrical T-shapes, extended T-shapes (or Y-shapes) with smaller α-subunit and bigger β-subunit facing opposite directions, and Γ-shapes (Fig. 5). Asymmetrical and extended T-shapes resemble the preactive conformation of IR with only one insulin molecule bound to the receptor (13, 33) (Fig. 5, images A 1–25, D16–20), whereas symmetrical T-conformation is close to the active conformation of the IR (Fig. 5, images A16, D21–25). Correspondingly, distribution of lengths of the ectodomain in T-shape has two peaks, 20 ± 1 nm and 26 ± 4 nm at pH 7.0 (Fig. 5*C*), and 22 ± 3 nm and 30 ± 1 nm at pH 9.0 (Fig. 5*F*) with the same height of 3.5 ± 0.5 nm (Fig. 5, *C* and *F*). The difference lies in relative positions of the subunits. In the symmetrical T-form of IRR, its subunits overlap in the middle, whereas L1C domains face outward. This configuration means that these domains in the AFM images are equal in size which is the case presented in Figure 5, images A16 and D21 to 25. We mostly observed it at pH 9.0, but on rare occasions, pH 7.0 also gave us such results. In the Y-shape, the IRR subunits do not overlap with each other as they do in the T-shape but rather unzip from the drop-like shape into opposite sides because their heights in the AFM images are the same as the height of the middle part (Fig. 5, images A3-5, A14, D9, D19, D22). Such a conformation is also detected for IR (34), where it indicates possible transient state between the Λ-shape with L1C domain contacting fibronectin domain and classical T-shape of IR. Γ-shape of IRR, similar to the active conformation of the IGF-1R, was detected only at pH 9.0 (Fig. 5, images D1-5). For this conformation, the length of the ectodomain part was 23 ± 1 nm and the height was 3.5 ± 0.5 nm. Thе value for the length is between that for the extended (second peak in Fig. 5, *C* and *F* for L_E_) and symmetrical T-shape (first peak in Fig. 5, *C* and *F* for L_E_) forms of the receptor. Two tyrosine kinase domains in all of these conformations were always visualized either as a single particle or a close dimer, indicating that this complex can be attributed to the active state of the receptor.

**Figure 6:**
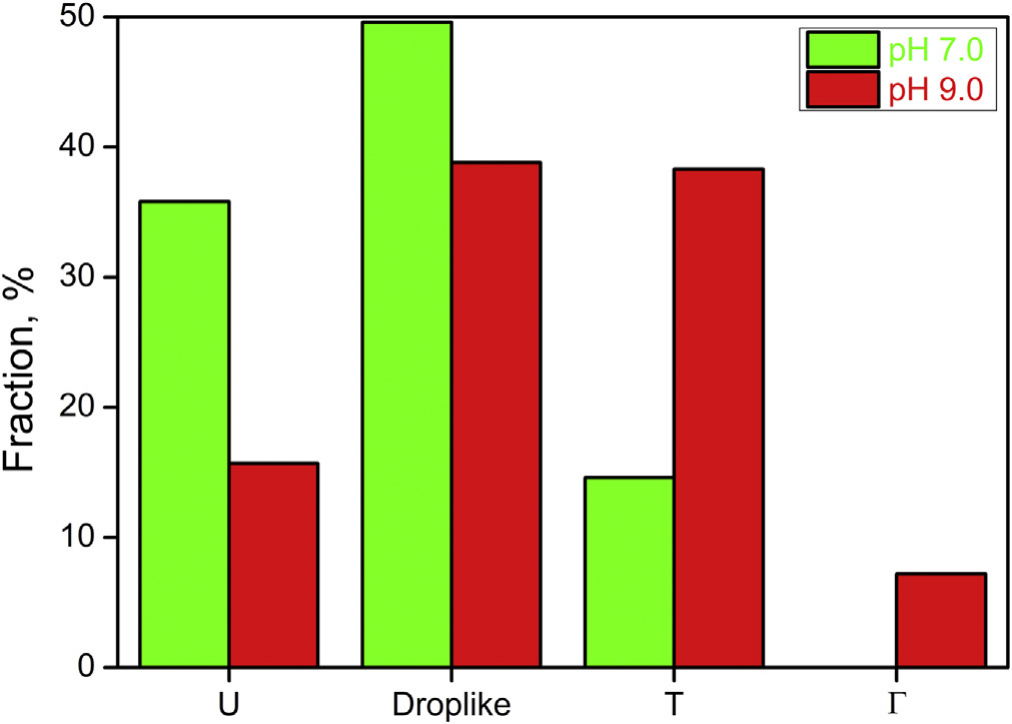
**Fraction of conformations of the ectodomain of the full-length IRR in micelles at pH 7.0 and pH 9.0.** Total number of analyzed IRR-micelle complexes was 250 for pH 7.0 and 338 for pH 9.0. IRR, insulin receptor-related receptor.

To quantify relative fractions of the observed conformations and their change with pH, we analyzed 250 IRR-micelle complexes at pH 7.0 and 338 complexes at pH 9.0 (Fig. 6). At both pH 7.0 and pH 9.0, the drop-like shape dominated; however, its fraction at neutral medium was 39%, whereas at pH 9.0, it reached 50%. Fractions of U-, T- and Γ-shaped conformations were significantly different in neutral and alkaline media. The amount of molecules with the U-shaped conformation was 36% at pH 7.0 and decreased more than twice, to 16% at pH 9.0. At the same time, the fraction of T-shapes of all types was 14% at pH 7.0, whereas at pH 9.0, it was 38%. The fraction of Γ-forms was visualized only at pH 9.0, where it was equal to 7%.

## Discussion

The insulin receptor mini family contains three RTKs among which IRR is the most enigmatic representative lacking structural data. Being a close homolog of other receptors from the IR family, IRR does not have a specific ligand and activates merely by an increase in the extracellular pH (35). Recently, we have shown that water soluble IRR ectodomain adopts a drop-like shape at neutral pH conditions, at which it is presumably inactive and preserves this state in an alkaline media, which activates the full-length IRR in cells (21). This shape is asymmetrical that is, however, not a unique feature of RTKs and may reflect a relative shift of the transmembrane segments in the full-length protein (8, 10, 36). It is also characterized by a shorter distance between the fibronectin domains of the two subunits (9, 13).

Here, we utilized high-resolution AFM to obtain data on structural characteristics of the full-length receptor at physiological conditions with permitted free movements of the protein. We established a set of criteria, which allowed selecting the proper conformation of the receptor molecules using micelles as beacons and performing repeating images to exclude contacting moving particles from the IRR structure. This method allowed us to get structural data on IRR. All particles selected as receptor molecules in micelles demonstrated the typical RTK structure with ectodomain and tyrosine kinase domains outside the micelle covering the transmembrane part of the receptor. We observed that solubilized IRR predominantly existed in the preactive drop-like conformation of the ectodomain which was of the same size and shape (Fig. 4) as the individual soluble IRR ectodomain studied by us previously (21). Together with this conformation for the full-length receptor, we also observed U-shape of the IRR ectodomain (Fig. 3) which was similar to inactive conformation of IGF-1R and IR. Thus, it might be considered to be the inactive state of the IRR. This statement is also supported by the fact that a fraction of U-shaped conformations decreased by more than twice in alkaline medium, which activates the receptor (35), compared with neutral pH conditions. Additionally, we observed symmetrical and asymmetrical T-shapes of the ectodomain of IRR as well as Γ-shaped conformation of the receptor. These conformations are analogous to the active states of IR and IGF-1R (13, 33, 34, 35).

In active conformations of IRR, tyrosine kinase domains were present as one particle (or in a very close proximity to each other), indicating possible activation. Despite the fact that some amount of such conformations was observed even at pH 7.0, it was at some basal level with the fraction of about 14%, which increased by three times at pH 9.0 (Fig. 6). Moreover, the number of symmetrical T-shapes dramatically increased at pH 9.0. The Γ-shaped IRR molecules were detected only in alkaline media.

**Figure 7:**
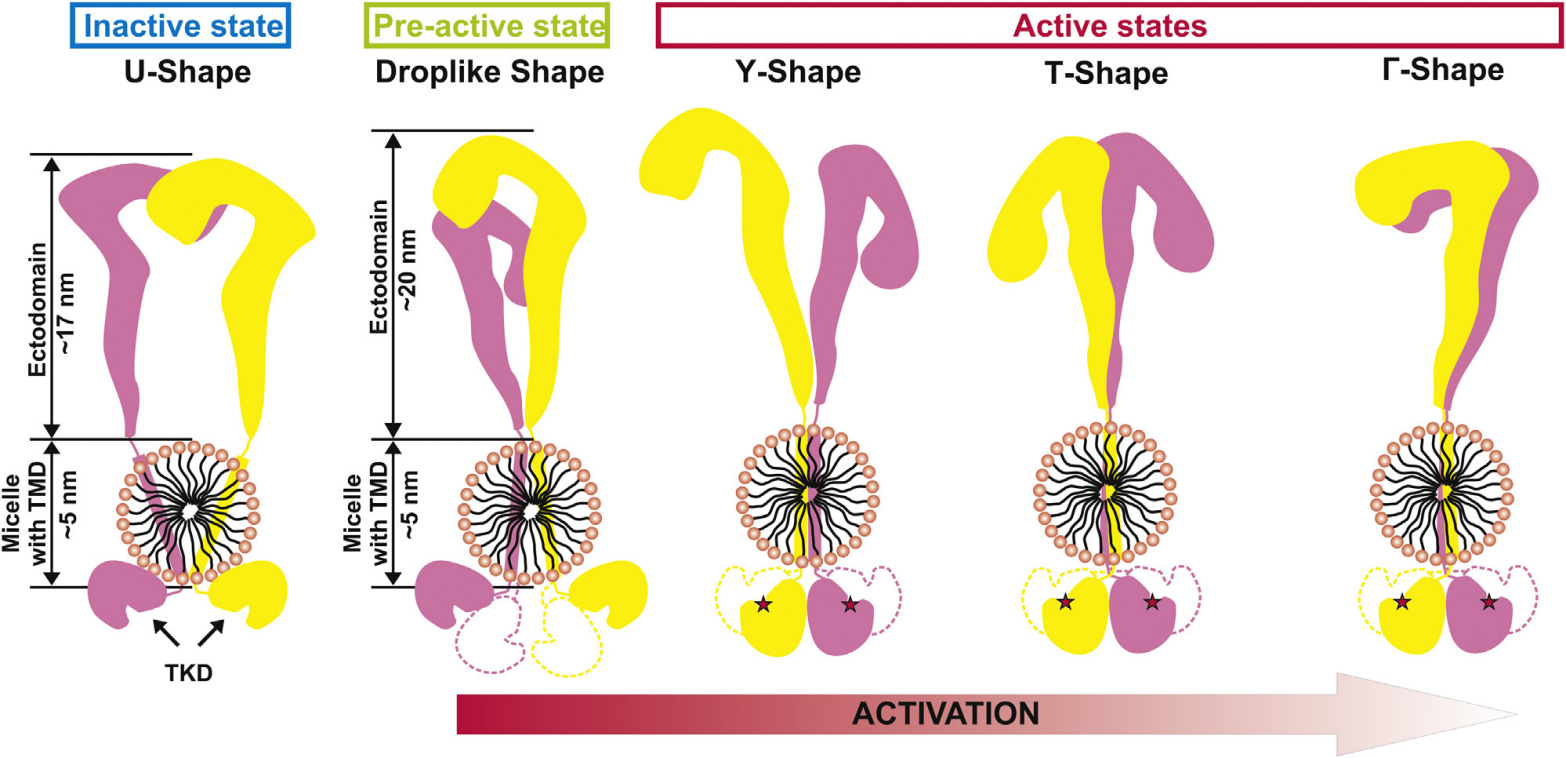
**Diagram showing observed IRR shapes and the predicted mode of its activation.** IRR, insulin receptor-related receptor; TMD, transmembrane domain; TKD, tyrosine kinase domain.

Our data suggest that full-length IRR has an inactive conformation which lies between the typical inactive Λ-shape of IR with the separation distance between subunits of about 12 nm (8) and inactive U-shaped conformation of IGF-1R where this distance is about 7 nm (11). In this conformation of IRR, its tyrosine kinase domains are almost unresolved from the micelle (Fig. 3) suggesting their tight attachment to its surface. On the contrary, in drop-like conformations, these domains are free to move and rarely join to form one dimer (Fig. 4). This fact indicates a possible existence of the preactive role of the drop-like conformation. Orphan nature of IRR suggests that it does not have specific proteinaceous endogenous ligands, which activate global rearrangements of the receptor dimer. Thus, if IRR predominantly exists in inactive conformation, it will hardly perform a transition to the active conformation merely by an increase in pH because it requires significant movement of the receptor subunits. Therefore, it is reasonable to hypothesize that IRR exists in cells bearing a more compact preactive drop-like conformation of its ectodomain (Fig. 7) that is ready to transition into the active form. This conformation is also observed for IGF-1R (12). Previous mutagenesis analysis revealed five conserved amino acid residues to be crucial for IRR activity (17). If IRR only has inactive U-shape, these amino acid residues would be turned outward facing the aqueous environment that is unlikely to regulate the IRR activity. Preactive drop-like shape of the ectodomain suggests that these five amino acid residues are located in the contact region between L1C and fibronectin domains within the IRR dimer explaining their role in the IRR activation (21). Moreover, transition to the active T-shaped or Γ-shaped symmetrical conformations should involve mutual shift of the subunits within the region of fibronectin domains and should involve the interface between L1C and fibronectin domains. Thus, these data support the suggested model of IRR activation from preactivated states into fully active T-shaped or Γ-shaped conformations.

Observed asymmetrical T-shaped (or Y-shaped) conformations correspond to the transient form of a single-liganded IR dimer (13, 33). Because IRR has no specific ligand for activation, we cannot distinguish these conformations into active or preactive states of the receptor especially because the position of tyrosine kinase domains is similar to symmetrical T-shaped conformation. The only exception is that the Γ-shape was detected only at pH 9.0. However, we hypothesize that the increase in extracellular pH should trigger rearrangements within the receptor dimer from inactive U-shape through the preactive drop-like shape into the asymmetrical Y-shape and ultimately the symmetrical T-shape or Γ-shape conformations in which tyrosine kinase domains dimerize and perform autophosphorylation. In fact, activation of IRR merely by the environmental pH suggests that transitions between possible preactive and active conformations of the receptor should occur continuously and follow some kind of a titration curve. It means that activation does not simply occur by a transition from a single inactive to a single active form but goes through an ensemble of conformations that are similar to active states of ligand-activated representatives of the insulin receptor family. This hypothesis correlates well with the described positive cooperativity of IRR alkali-induced activation (16).

## Conclusions

A general mechanism of RTK activation consists of dimerization following their ligand binding. Highly homologous IR, IGF-IR, and IRR are an exception because they are expressed as dimers and, therefore, have to undergo a conformational transition to get activated. For IR and IGF-IR, this transition requires their agonist binding, whereas IRR is an orphan receptor having no proteinaceous ligands and requiring a pH change for activation. Thus, IRR is a convenient target for structural studies of its activation mechanism.

Here, we developed a new technique to obtain structural data on the full-length IRR in the native physiological conditions by using high-resolution AFM. We detected four IRR conformations which represent different stages of its activation. According to biochemical data, IRR is inactive in neutral media. In this preparation, AFM revealed two predominant conformations, where one is symmetrical and highly similar to the inactive Λ/U-shape of IR and IGF-1R ectodomains (11, 13, 33), whereas the second is drop-like and asymmetrical resembling the IRR ectodomain in solution (21). The intracellular catalytic domains of IRR in the inactive receptor forms are not complexed, indicating the absence of IRR autophosphorylation in neutral media. At pH 9.0, two presumably active IRR conformations are detected that have Γ- and T-shapes resembling ligand-activated IR and IGF-1R (13, 33). Both activated IRR forms demonstrate complexing of their intracellular catalytic domains responsible for autophosphorylation. The existence of two active IRR forms correlates well with the previously described positive cooperativity of the IRR activation (16).

In conclusion, our data provide structural insights into the molecular mechanisms of alkali-induced IRR activation in solution that could be valuable to interpreting results of IR and IGF-1R structural studies.

## Experimental procedures

### Expression and purification of the full-length IRR

Human IRR cDNA was cloned into the pcDNA3.1 vector resulting in a construct consisting of the human IRR followed by a C-terminal 6хHIS tag.

The receptor was produced in HEK293 cells (ATCC CRL-1573) that were grown at 37 °C, 5% CO_2_ in Dulbecco's modified Eagle's medium (Paneco) supplemented with 10% fetal bovine serum (Gibco), 1% of penicillin/streptomycin (Paneco), and 2 mM L-glutamine (Paneco) (37).

The cells were transiently transfected at approximately 80% confluency with plasmid pcDNA-IRR-HIS (50 μg per 175 cm^2^ monolayer surface area) using the FuGENE 6 reagent (Promega) as described in (38). Upon transfection, the cells were maintained for 72 h at 37 °C, 5% CO_2_. Cells were harvested, washed once with Versen's solution, centrifuged at 3000 g for 10 min, and either used immediately or stored at −80 °C.

Cells transfected with IRR-HIS were thawed and resuspended in buffer [20 mM sodium phosphate, pH 7.4, 500 mM NaCl, 20 mM imidazole, 5% glycerol, 1 mM PMSF, protease inhibitor cocktail (P8340 Sigma-Aldrich), 1% DDM] on ice. Resuspended cells were disrupted using a tip sonicator (Sonics Vibra-Cell) at 4 x 15 s impulse on, 60 s impulse off, 50% output.

Cell lysates were cleared by centrifugation at 11,000 g for 90 min at 4 °C, and the cleared supernatants were applied to a Ni Sepharose 6 Fast Flow column equilibrated with wash buffer (20 mM sodium phosphate, pH 7.4, 500 mM NaCl, 20 mM imidazole, 5% glycerol, 0.05% DDM). The column was washed with 20 column volumes of wash buffer, and bound sample was eluted with wash buffer containing 500 mM imidazole. Eluted fractions were dialyzed overnight at 4 °C to 20 mM sodium phosphate, pH 7.4, 150 mM NaCl, 5% glycerol, 0.05% DDM.

Dialyzed eluate was applied to a Cyanogen bromide-activated-Sepharose 4B column (Sigma-Aldrich) with linked monoclonal antibodies 4D5 (30, 31) equilibrated in buffer 20 mM sodium phosphate, pH 7.4, 150 mM NaCl, 5% glycerol, 0.05% DDM. The column was washed with 20 column volumes of wash buffer, and bound sample was eluted in buffer 20 mM sodium phosphate, pH 7.4, 3 M MgCl_2_, 5% Glycerol, 0.05% DDM. Eluted fractions were dialyzed overnight at 4 °C to 20 mM sodium phosphate, pH 7.4, 150 mM NaCl, 5% glycerol, 0.05% DDM.

### Autophosphorylation *in vitro*

After purification, MgCl_2_ and Na_3_VO_4_ were added to the protein solution of a final concentration of 15 mM and 1 mM, respectively. Two samples of the protein solution were supplemented with 1M Tris-HCl, pH 7.4 or 9.0, and incubated for 30 min on ice. Furthermore, ATP was added to the final concentration of 100 nM, and samples were placed at 25 °C for 5 min. To stop autophosphorylation reaction, 5X SDS loading buffer was added.

### Western blot

SDS-PAGE in 8% gel followed by Western blotting was carried out according to the standard protocols as described in (39). Proteins were transferred onto a nitrocellulose membrane for 1.5 h at 250 mA current. The nonspecific sorption of proteins was prevented by incubation of the membrane for 1 h in TBST buffer (50 mM Tris-HCl, pH 8.0, 150 mM NaCl, 0.05% Tween 20) containing 5% of skim milk powder. Then, the membrane was incubated with solution of primary antibodies in TBST (1:2000).

The total amount of the IRR was determined using polyclonal rabbit anti-C-IRR antibodies against the C-terminal cytoplasmic domain (961–1297 amino acid residues). The anti-pIRR antibodies were raised against KLH-coupled peptide CGMTRDVpYETDpYpYRKGGKGL from the activation loop of IRR and used to measure the level of phosphorylation (16). Then, the membrane was washed from primary antibodies, and the secondary donkey antibodies against rabbit immunoglobulins IgG conjugated with horseradish peroxidase (Jackson ImmunoResearch) were added. The bands were visualized by treatment with the luminescent substrate SuperSignal West Pico (Pierce) on a Fusion Solo device (Vilber Lourmat).

### Atomic force microscopy

50 nM IRR aliquots of stock solution in 150 mM NaCl, 20 mM sodium phosphate, 5% glycerol buffer (pH 7.0 or pH 9.0) containing 0.05% DDM were diluted 10 to 20 times with the same buffer. 100 μl protein solution droplet was deposited onto a mica surface for 20 min and after that rinsed off with the same buffer to remove unbound particles. Samples of IRR were imaged in tapping mode using a Multimode Nanoscope V AFM (Bruker) equipped with the J type scanner and an electrochemical fluid cell. To obtain high resolution, ultrasharp SNL-10 cantilevers with a nominal spring constant of 0.06 N/m and a tip radius of approximately 2 nm (Bruker) were used, because radius of the cantilever determines the lateral resolution of AFM, while its height (z-axis) resolution is about 0.1 nm. Scan rate was typically 2 Hz. Image processing was performed using the FemtoScan Online software (Advanced Technologies Center).

## Data availability

All data are presented in the paper. Raw data are available upon request (olegbati@gmail.com).

## Supporting information

This article contains supporting information.

## Conflicts of interest

The authors declare no conflict of interest. The founding sponsors had no role in the design of the study; in the collection, analyses, or interpretation of data; in the writing of the manuscript; and in the decision to publish the results.
