# Supplementary material for: Activity-dependent conformational transitions of the insulin receptor–related receptor
Source: J Biol Chem. 2021 Mar 11;296:100534. doi: 10.1016/j.jbc.2021.100534 (PMC8058561; doi:10.1016/j.jbc.2021.100534)
Supplement: Figure S1 [file mmc1.pdf]

Supporting Material

Activity-dependent conformational transitions of the insulin receptor-related receptor

Oleg V. Batishchev [1*#], Natalia V. Kuzmina [1#], Andrey A. Mozhaev [2,3], Alexander S. Goryashchenko [2], Ekaterina D. Mileshina [2], Alexander N. Orsa [2], Eduard V. Bocharov[2,4], Igor E. Deyev [2], Alexander G. Petrenko[2]

[1] A.N. Frumkin Institute of Physical Chemistry and Electrochemistry, Russian Academy of Sciences, Moscow, Russia;
[2] Shemyakin–Ovchinnikov Institute of Bioorganic Chemistry, Russian Academy of Sciences, Moscow, Russia
[3] Shubnikov Institute of Crystallography of Federal Scientific Research Centre "Crystallography and Photonics", Russian Academy of Sciences, Moscow, Russia
[4] Moscow Institute of Physics and Technology, Dolgoprudniy, Moscow Region, Russia

* Correspondence: olegbati@gmail.com; Tel.: +7-495-955-4585

[#] These authors contributed equally to the work

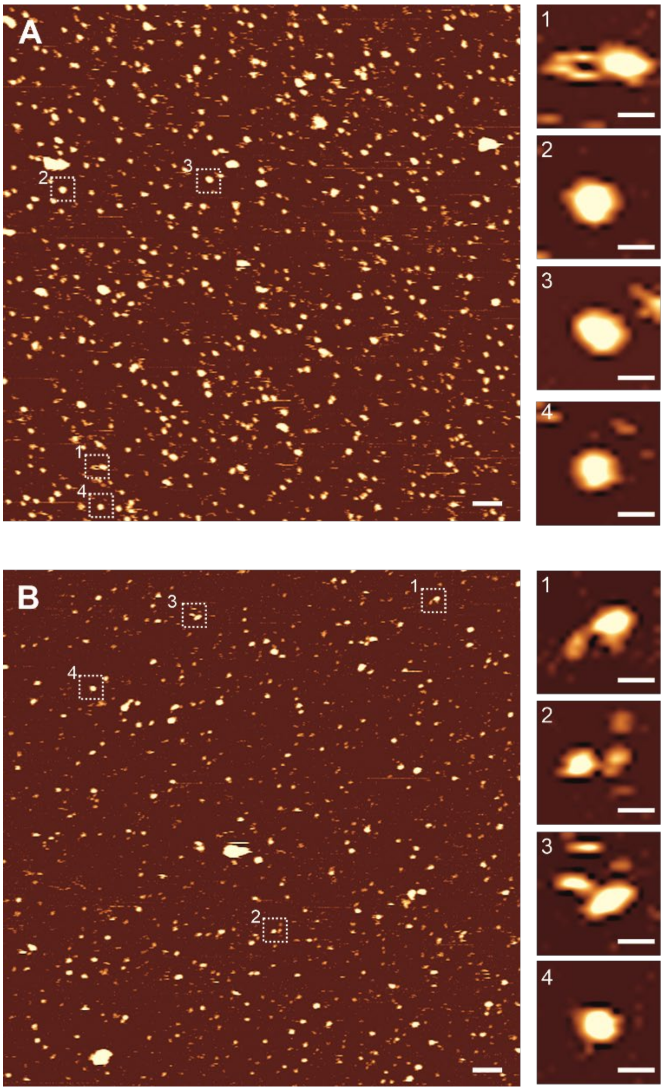

Figure S1. Examples of large-scale AFM images of the full-length IRR in micelles used for selection of typical conformations. **A** – pH 7.0, **B** – pH 9.0. A 2-4 and B 4 represent empty micelles. Scale bars are 100 nm for the main images and 20 nm for zoom-ins. Z-scale is 4 nm.
